# Comparison of performance in a four year graduate entry medical programme and a traditional five/six year programme

**DOI:** 10.1186/s12909-014-0248-3

**Published:** 2014-12-10

**Authors:** Annette T Byrne, Richard Arnett, Tom Farrell, Seamus Sreenan

**Affiliations:** Royal College of Surgeons in Ireland, Graduate Entry Programme, Reservoir House, Ballymoss Road, Dublin, 18 Ireland; Royal College of Surgeons in Ireland, Quality Enhancement Office, 123 St. Stephen’s Green, Dublin, 2 Ireland

**Keywords:** Graduate entry medicine outcomes

## Abstract

**Background:**

In 2006 the Royal College of Surgeons in Ireland, (RCSI), introduced the first four year Graduate Entry Programme (GEP) in medicine in Ireland in line with national policy to broaden access to medical education. One concern considered at the time, was whether the GEP students could be trained to the same standard as their undergraduate Direct Entry Programme (DEP, five/six year duration) counterparts in the shorter time frame. Since students from both cohorts undertake the same examinations in the final two years, it is possible to directly compare GEP vs DEP outcomes. The primary aim of the current study was to analyse the comparative performance of GEP and DEP students undergoing these examinations between 2008 and 2013.

**Methods:**

Scores from five assessments performed during the final two years were transformed to z scores for each student and 4 scores for the penultimate year were summed to create a unit weighted composite score. The resultant scores for each of the two years were used to assess the comparative performance of GEP vs DEP cohorts and to perform sub-cohort analyses of GEP outcomes.

**Results:**

In all cohorts/years examined, evidence demonstrated significantly better assessment outcomes for the GEP group for the final two years’ examinations as compared with the DEP group. In all but one cohort examined, this advantage was retained when nationality factors were excluded. Further analyses showed no difference in outcomes between GEP students having science vs. non-science backgrounds and/or between those from EU vs non-EU backgrounds. Finally, data suggested weak correlations between total composite scores and entry scores in American (r = 0.15) and Australian (r = 0.08) medical school admissions tests.

**Conclusions:**

We have shown for the first time in Ireland, that graduate-entry students perform at least as well, or even better, than a corresponding undergraduate-entry group. Moreover, having a scientific background on entry to the GEP confers no advantage in final assessments. These data provide evidence of the viability of the graduate entry route into medical education in Ireland.

## Background

Traditionally, most students entering medical school in Ireland did so directly upon leaving secondary education, entering a programme which took 5 or 6 years to complete. At the Royal College of Surgeons in Ireland (RCSI) students undertaking a 5 year programme complete Junior Cycle (3 semesters), Intermediate Cycle (3 semesters) and Senior Cycle (4 semesters). Students without a strong grounding in basic sciences also undertake a 2 semester Foundation Year before entering the Junior Cycle thus taking six years to complete the Programme. Irish and other EU students access the traditional Medicine programme via the same national Central Applications Office (CAO) as other university medicine programmes in Ireland. Students from outside the EU are subject to a rigorous application and interview process. [N.B for the purposes of this paper, the traditional five/six year programme is referred to as the Direct Entry Programme (DEP)]. In 2006 RCSI, Ireland’s largest medical school, introduced a four year Graduate Entry Programme (GEP) in medicine, the first of its kind in Ireland. This initiative was developed in line with national policy with an aim of broadening access to medical education in Ireland. As GEP students are expected to acquire the same competencies as students undertaking the programme in 5 or 6 years, the programme is more intensive. On successful completion of two preclinical years, GEP students join their DEP colleagues for the final two years of the classical Medicine Programme. To be eligible for entry to the GEP, applicants must hold a level 8 honours degree with a minimum 2:1 honours classification (in any subject). Applicants must also achieve a competitive score on either the Graduate Medical School Admission Test (GAMSAT-EU students and non-EU students) or the Medical College Admission Test (MCAT – non-EU students). The latter criteria are norm-referenced such that applicants with the highest scores have priority for admission. Irish and other EU applicants apply through the CAO in the same way as their DEP counterparts; non-EU applicants are subject to an interview process in addition to the academic criteria.

One of the concerns, given the shorter duration of training in the GEP, was that the students in the two programmes may not be of an equivalent standard. However, over the past 7 year period, the perception has been that GEP students have performed as well as their DEP counterparts, although no statistical metrics had to date been applied to formally establish these observations. Since the students undertake the same final exit examinations, it is possible to compare the performance of the students in the GEP to that of those in the DEP. We hypothesized that the performance of the students in GEP would be equivalent to that of their DEP counterparts. Thus, the primary aim of the current study is to analyse the comparative performance of four cohorts of GEP and DEP students undertaking the examinations in the final two years of the Medicine Programme between 2009 and 2013. We performed additional analyses of the GEP cohorts to assess impact of scientific background, nationality and MCAT/GAMSAT entry test scores on outcomes in the clinical years’ examinations.

## Methods

For the last two years of GEP and DEP (Senior Cycle) students are amalgamated into a single group for Senior Cycle 1 (SC1) and Senior Cycle 2 (SC2). During SC1, students complete senior clinical rotations in Psychiatry, General Practice, Obstetrics & Gynaecology, Paediatrics, and Clinical Medicine/Surgery. SC2 consists of further clinical training in Medicine and Surgery. Assessment formats used during the Senior Cycle include a variety of written and observed formats including (but not limited to) MCQs, modified essays, case presentations, Objective Structured Clinical Examinations, observed long case examinations, data interpretations and portfolios. On completion of all SC1 assessments, students obtain four overall scores for Psychiatry, Obstetrics & Gynaecology, Paediatrics, and Clinical Medicine (including Surgery and General Practice). The initial number of individual students was 937. For inclusion in this study, students were required to have a complete set of five scores (i.e. four scores for SC1 and 1 score for SC2). Only 1 student did not meet these criteria and was excluded leaving 936 individual students (DEP n = 703, GEP n = 233). The total numbers of students included in each cohort/year is shown in Table 1. All scores were transformed to z scores and the z scores for each student for SC1 were summed to create a unit-weighted composite score. This resulted in a single score for each student for SC1 and a single score for each student for SC2.

**Table 1:** **Total numbers of DEP and GEP students included in each cohort over a four year period**

**Cohort (Year)**	**DEP (n)**	**GEP (n)**
Cohort 1 SC1 (2008-2009)	195	49
Cohort 1 SC2 (2009-2010)	194	49
Cohort 2 SC1 (2009-2010)	167	62
Cohort 2 SC2 (2010-2011)	163	62
Cohort 3 SC1 (2010-2011)	164	60
Cohort 3 SC2 (2011-2012)	155	60
Cohort 4 SC1 (2011-2012)	191	62
Cohort 4 SC2 (2012-2013)	185	60

Data were assessed for normality using the Shapiro-Wilk test. Comparative data which did not show evidence of significant departure from normality were analysed using independent t-tests, and data which showed significant departures from normality were compared using non-parametric Mann-Whitney-Wilcoxon tests. Effect sizes for non-parametric data uses r [1] and effect sizes for parametric data was estimated using Cohen’s d. Correlation was measured using Pearson’s r.

Ethical approval to perform these studies was obtained from the Royal College of Surgeons Human Research Ethics Committee (HREC).

## Results

### Comparative performance of GEP vs DEP cohorts in SC1 and SC2

**Figure 1:**
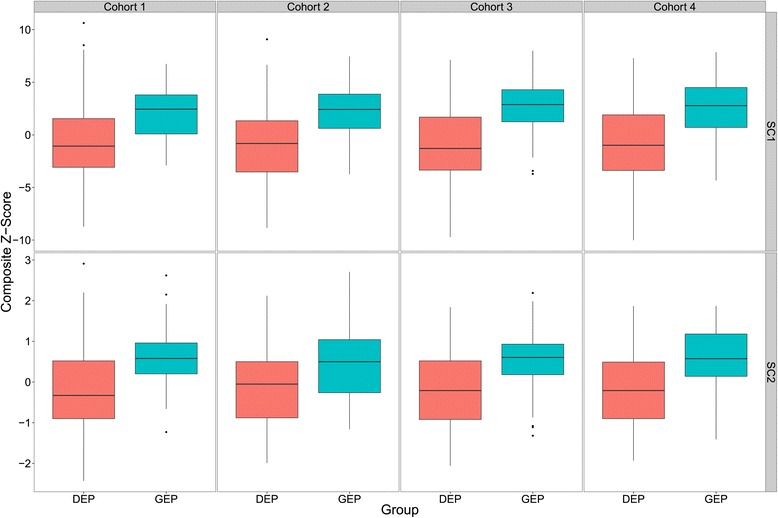
**Comparative composite z-score distributions for four cohorts of DEP and GEP students in SC1 and SC2 examinations.** The upper and lower margins of the box represent the 75th and 25th quartiles of the distribution (the interquartile range) and the thick horizontal line within the box defines the median value. The vertical lines above and below the box represent the range of the data up to 1.5 times the interquartile range above and below the 75th and 25th quartiles. Values that are more than 1.5 times greater or less than the 75th and 25th quartiles are defined as outliers and are shown as dots.

Assessing data for eligible students for all Cohort/Years, all but Cohort 2 SC1 showed evidence of departure from a normal distribution and thus further comparisons were performed using non-parametric Mann-Whitney-Wilcoxon tests. Comparative boxplots of data for each Cohort/Year are shown in Figure 1.

The results of Mann-Whitney-Wilcoxon tests are shown in Table 2. All cohorts/years examined provide evidence to suggest significantly better outcomes for the GEP cohorts for both SC1 and SC2 examinations with moderate effect sizes.

**Table 2:** **Summary statistics for non-parametric comparison of composite z-scores for four cohorts of DEP and GEP students**

**Cohort (Year)**	**DEP Median**	**GEP Median**	**DEP IQR**	**GEP IQR**	**U**	**p**	**Effect (r)**
Cohort 1 SC1 (2008-2009)	-1.07	2.44	4.64	3.71	2390	<0.01	0.35
Cohort 1 SC2 (2009-2010)	-0.33	0.58	1.42	0.76	2464	<0.01	0.33
Cohort 2 SC1 (2009-2010)	-0.82	2.42	4.87	3.25	2449	<0.01	0.40
Cohort 2 SC2 (2010-2011)	-0.05	0.50	1.38	1.31	3248	<0.01	0.28
Cohort 3 SC1 (2010-2011)	-1.29	2.88	5.04	3.05	2000	<0.01	0.45
Cohort 3 SC2 (2011-2012)	-0.21	0.61	1.44	0.75	2426	<0.01	0.37
Cohort 4 SC1 (2011-2012)	-0.99	2.78	5.29	3.80	2728	<0.01	0.40
Cohort 4 SC2 (2012-2013)	-0.21	0.58	1.39	1.04	2924	<0.01	0.35

Data shown include median scores, interquartile ranges (IQR), the test statistic U, the p-value and effect size (r).

**Figure 2:**
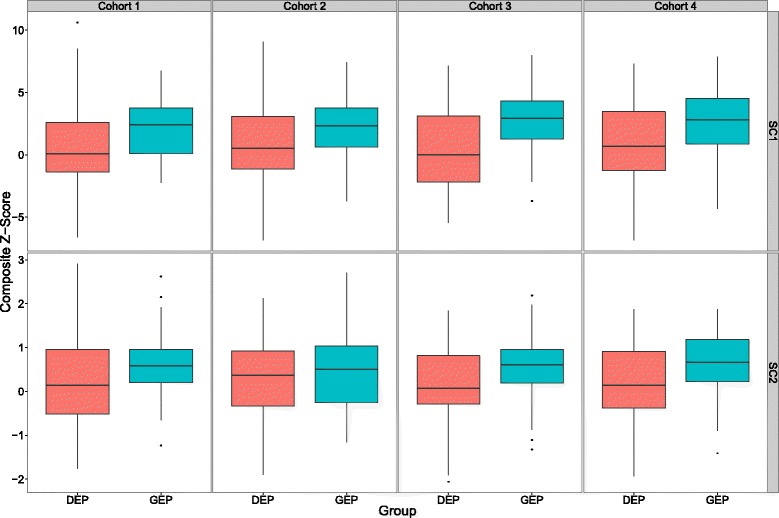
**Comparative composite z-score distributions for four cohorts of DEP and GEP Irish/UK and North American students in SC1 and SC2 examinations.** The upper and lower margins of the box represent the 75th and 25th quartiles of the distribution (the interquartile range) and the thick horizontal line within the box defines the median value. The vertical lines above and below the box represent the range of the data up to 1.5 times the interquartile range above and below the 75th and 25th quartiles. Values that are more than 1.5 times greater or less than the 75th and 25th quartiles are defined as outliers and are shown as dots.

As the GEP group is comprised largely of Irish/UK (49%) and North American students (47%) (Table 3), these nationality groupings in each cohort were further assessed to apply a more appropriate comparison (Figure 2 and Table 4). Data were largely normally distributed and thus comparisons were made using independent t-tests. With the exception of Cohort 2 (SC2), significantly better outcomes for the GEP groups for both SC1 and SC2 examinations were still evident, when comparisons were made between the GEP and DEP using only the major nationality groupings. The effect size (d) across cohorts was in the moderate to high range.

**Table 3:** **Nationalities of DEP and GEP Senior Cycle students between 2008 and 2013**

**Nationality**	**DEP(n)**	**GEP (n)**
Asia	149	3
Europe	26	3
Ireland/UK	171	115
Middle East	176	2
North America	151	110
Other	30	0

Data shown include mean scores, standard deviations (SD), the test statistic (t), degrees of freedom (df), the p-value and effect size (Cohen’s d).

**Table 4:** **Summary statistics for parametric comparison of composite z-scores for four cohorts of Irish/UK and North American DEP and GEP students**

**Cohort (Year)**	**DEP Mean**	**GEP Mean**	**DEP SD**	**GEP SD**	**p**	**t**	**DF**	**Effect(d)**
Cohort 1 SC1 (2008-2009)	0.69	2.07	2.29	3.45	<0.01	-2.84	125.69	0.47
Cohort 1 SC2 (2009-2010)	0.22	0.62	0.74	1.03	0.01	-2.60	119.20	0.44
Cohort 2 SC1 (2009-2010)	0.83	2.27	2.68	3.21	<0.01	-2.81	129.86	0.49
Cohort 2 SC2 (2010-20 11)	0.28	0.46	0.84	0.91	0.26	-1.13	126.65	0.20
Cohort 3 SC1 (2010-2011)	0.44	2.78	2.49	3.16	<0.01	-4.77	129.93	0.82
Cohort 3 5C2 (2011-2012)	0.08	0.62	0.77	0.92	<0.01	-3.66	128.94	0.64
Cohort 4 SC1 (2011-2012)	0.82	2.51	2.58	3.33	<0.01	-3,43	142.40	0.57
Cohort 4 SC2 (2012-2013)	0.25	0.61	0.69	0.88	<0.01	-2.73	138.56	0.45

Data shown include mean scores, standard deviations (SD), the test statistic (t), degrees of freedom (df), the p-value and effect size (Cohen’s d).

### Sub-cohort analyses of GEP SC outcomes

In order to further assess outcomes of GEP students in SC clinical examination years the following additional analyses were performed in this cohort:

#### GEP students background: science v non science

**Figure 3:**
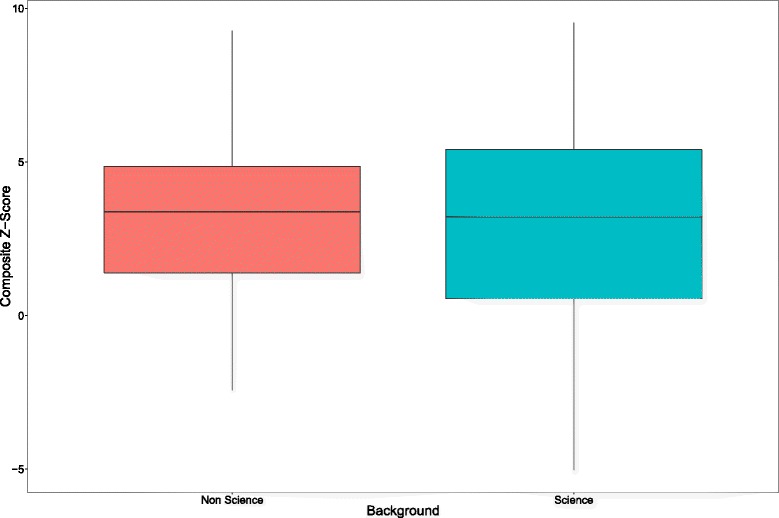
**Total composite z-score distribution of GEP students from Science and Non-Science backgrounds over four years.** The upper and lower margins of the box represent the 75th and 25th quartiles of the distribution (the interquartile range) and the thick horizontal line within the box defines the median value. The vertical lines above and below the box represent the range of the data up to 1.5 times the interquartile range above and below the 75th and 25th quartiles. Values that are more than 1.5 times greater or less than the 75th and 25th quartiles are defined as outliers and are shown as dots.

A small proportion of students on the GEP have non-science qualifications (e.g. English, History, Law and Languages) and thus it is appropriate to investigate whether this cohort of students performs differently than those that have progressed through more traditional scientific disciplines (e.g. Life Sciences, Physical Sciences and Computational Sciences). Due to limited student numbers, the data for all cohorts/years was combined into two categories, ’Science’ (n = 202) and ‘Non Science’ (n = 29). The comparative total composite score distribution is shown in Figure 3. There was no evidence to suggest a difference in SC outcomes between the Science vs Non Science groups (t = 0.56, df = 37.89, p = 0.58).

#### GEP students nationality: Irish/UK V North America

**Figure 4:**
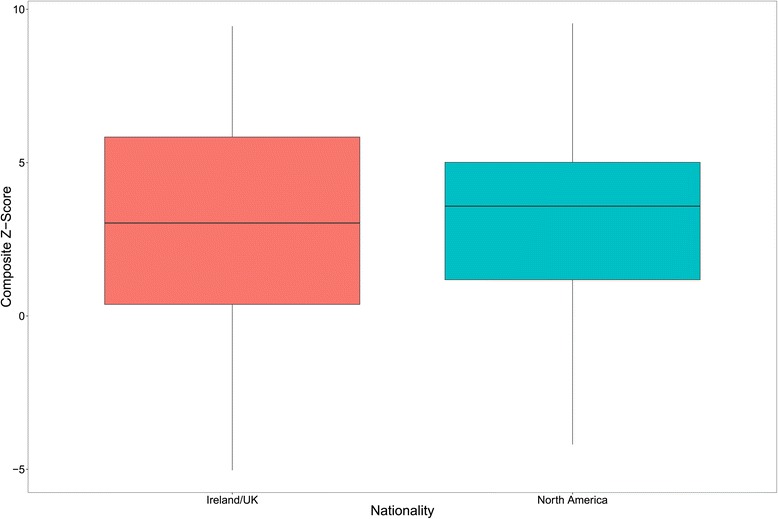
**Comparative total composite z-score distributions for Irish/UK and North American GEP students over four years.** The upper and lower margins of the box represent the 75th and 25th quartiles of the distribution (the interquartile range) and the thick horizontal line within the box defines the median value. The vertical lines above and below the box represent the range of the data up to 1.5 times the interquartile range above and below the 75th and 25th quartiles. Values that are more than 1.5 times greater or less than the 75th and 25th quartiles are defined as outliers and are shown as dots.

As the GEP cohort each year consists of a roughly even split between EU, predominantly Irish/UK students, and North American students, it is also useful to ascertain if there is any evidence to suggest an overall difference in performance between these two groups. Again, the data for all cohorts/years were combined into two categories, ‘Irish/UK’ (n = 114) and ‘North American’ (n = 109). The comparative total composite score distribution is shown in Figure 4. There was no evidence of any difference in the composite scores of Irish/UK vs North American GEP students (t = 0.46, df = 37.78, p = 0.65).

#### GAMSAT & MCAT as predictors of outcome

Most Irish/UK students use the GAMSAT to fulfil entry requirements and most North American students use the MCAT. It is pertinent to ascertain if there is any evidence to suggest that these different entry tests have any predictive value for GEP students in relation to total composite scores in the final two years of the Medicine programme. There was a very weak though positive correlation between total composite scores and MCAT entry scores (r = 0.15) and an even weaker correlation between total composite scores and GAMSAT entry scores (r = 0.08).

## Discussion

There has been a substantial increase in the number of graduate-entry medical courses in Ireland, the UK and Australia over the last 10 years. Graduate entry to medicine creates greater diversity among applicants [2,3], and admits students who are generally older, more mature and academically proven [4-8]. In recent years an increasing number of studies have emerged to provide data comparing clinical (and pre-clinical) examination outcomes for graduate-entry vs undergraduate-entry students.

Recent evidence suggests that graduate-entry medical students may have a marginal academic performance advantage over undergraduate-entry students in a pre-clinical curriculum in both bioscience knowledge and clinical skills assessments [9]. In this study, graduate entrants performed consistently, but only marginally, better than undergraduate entrants on both bioscience knowledge and clinical assessments.

A number of studies have also compared graduate and undergraduate medical students on clinical assessments. Calvert *et al*.’s [10] findings suggested that graduate-entry students performed better than mainstream students in clinical assessments. In this study, the academic performance of graduate-entry medical students at the University of Birmingham was shown to be better than that of undergraduate-entry medical students, with graduate-entry students significantly more likely to achieve honours degrees.

Interestingly, Manning and Garrud [11] described worsening performance for graduate-entry students in knowledge assessments over the duration of the course. Mean performance on clinical assessments showed a significant overall difference, made up of lower performance on 4 of 5 knowledge-based examinations by the graduate entry group, but similar levels of performance on all the skills-based and attitudinal assessments. The pattern was that of two groups diverging over time, as students progressed through the shared clinical phases. Thus, graduate-entry medical students were better than undergraduate-entry medical students in clinical phase 1, they then deteriorated and performed worse than the undergraduate cohort through most of clinical phase 2 and clinical phase 3 years. Several hypotheses were put forward by the authors to explain these findings. Firstly, poorer performance, especially in a number of knowledge-based assessments represents a genuine trend that reflects weaker prior educational attainment. This theory was strengthened by the fact that the somewhat poorer performance was seen in knowledge-based assessments (typical of prior educational experience) but not in skill-based assessments. The second possibility was that differences in performance were related to the differing demographic profile of the graduate-entry vs undergraduate-entry student streams, the most prominent being the higher age of the graduate entry cohort and the lower proportion of females.

Shehmar *et al*. [12] compared performance in clinical examinations midway through clinical training, finding that undergraduate-entry students achieved better results than graduates. By the end of training, however, graduate and undergraduate entrants did not differ on final assessments. This was the first large-scale UK study to compare the performance of graduate-entry and school-leaver medical students following the same clinical curriculum and using the same assessments. Graduate-entry students were seen to perform as well as undergraduates in final examinations despite lower A-level grades and a shorter 4-year accelerated course. Most recently, Reid et al, [6] showed that graduate-entry and undergraduate-entry medical students undertaking an identical clinical curriculum performed similarly on academic assessments of clinical skills. When compared with other studies [9,12], the latter further suggests that any academic advantage conferred by a prior degree may be limited to the pre-clinical phase of the course.

Herein we have, for the first time in Ireland, objectively analysed the comparative performance of cohorts of graduate-entry and undergraduate-entry medical students. Examination outcomes were compared for students undergoing the final two (clinical) years of the Medicine Programme at RCSI.

Firstly, in all cohorts/years examined, evidence was provided to demonstrate significantly better assessment outcomes for the GEP group for both SC1 and SC2 examinations as compared with the DEP group. In all but one cohort examined, this advantage was retained when nationality factors were excluded. With the exception of the Manning and Garrud [11] findings discussed above, these data are consistent with several studies now suggesting that graduate-entry medical students perform at least as well, or even better, than a corresponding undergraduate-entry group [6,10,12].

Our further analyses demonstrated no evidence to suggest a difference in SC outcomes between GEP students having science vs non-science backgrounds (although the small numbers of non-science students limits the power of this comparison). These findings are also consistent with the majority of studies that found little or no difference in the performance by medical students from various academic backgrounds; e.g. [13-15] or, where differences did occur, a temporary advantage for those with science background e.g. [15]. It is worth noting that this lack of difference may be somewhat artificial, as no matter what academic background students come from, they have all demonstrated a high level of basic science ability by scoring highly on the MCAT or GAMSAT assessments where the science sections may be heavily weighted. Furthermore, there was no evidence of any difference in the composite scores of Irish/UK vs North American GEP students when z score outcomes for SC 1 and SC 2 examinations were cumulatively assessed over the four-year study period.

Finally, we assessed whether performance in the GAMSAT/MCAT entry tests had predictive value for GEP student performance when assessed by total composite scores achieved in the final two clinical years of the Medicine programme. Data suggested a weak positive correlation between total composite scores and MCAT entry scores (r = 0.15) and an even weaker correlation between total composite scores and GAMSAT entry scores (r = 0.08). It is worth remembering that these students are picked from the top of an ability group of applicants and are thus reasonably homogenous. It is possible that the range of MCAT/GAMSAT scores is too attenuated to demonstrate a relationship.

## Conclusions

In conclusion, our findings demonstrate for the first time within the context of an Irish medical school that graduate-entry students perform at least as well, or even better, than a corresponding undergraduate-entry group. Moreover, having a scientific background at time of entry to the GEP confers no significant advantage in final year clinical assessments. These data provide evidence of the viability of the graduate entry route into medical education in Ireland.

## Abbreviations

RCSI: Royal College of Surgeons in Ireland
GEP: Graduate entry programme
DEP: Direct entry programme
SC1: Senior Cycle 1
SC2: Senior Cycle 2
CAO: Central Applications Office
GAMSAT: Graduate Australian Medical School Admission Test
MCAT: Medical College Admission Test
